# Supplementary material for: Anti-inflammatory activity of lefamulin versus azithromycin and dexamethasone in vivo and in vitro in a lipopolysaccharide-induced lung neutrophilia mouse model
Source: PLoS One. 2021 Sep 29;16(9):e0237659. doi: 10.1371/journal.pone.0237659 (PMC8480901; doi:10.1371/journal.pone.0237659)
Supplement: S1 Appendix — (PDF) [file pone.0237659.s001.pdf]

S1 Appendix

Supplementary methods

Cytokine and chemokine levels in J774.2 mouse macrophage cell supernatant

Treatment and stimulation of J774.2 cells

J774.2 mouse macrophages (ECACC, Sigma-Aldrich Chemie GmbH, Munich, Germany) were grown in Dulbecco's Modified Eagle Medium (DMEM; Thermo Fisher Scientific, Waltham, MA, USA) supplemented with 10% fetal bovine serum (FBS; BioWest LLC, Riverside, MO, USA). A day before the experiment, J774.2 cells were seeded (1 mL [300,000 cells] per well) in a 24-well plate and incubated overnight at 37°C (5% CO_2, 95% humidity). On the day of the experiment, the media was aspirated, and 640 µL of fresh DMEM + 10% FBS was added to each well.

Lefamulin (BC-3781.Ac; Nabriva Therapeutics, Vienna, Austria) and azithromycin (BC-1024; Nabriva Therapeutics) were prepared in 3% dimethyl sulfoxide (DMSO; Sigma-Aldrich Chemie GmbH) in DMEM + 10% FBS at 10× concentrations of 0.3, 1, 3, 10, 30, 100, 300, and 1000 µM; dexamethasone (Sigma-Aldrich Chemie GmbH) was prepared in 3% DMSO in DMEM + 10% FBS at 10× concentrations of 0.003, 0.01, 0.03, 0.1, 0.3, 1, 3, and 10 µM. To each well, 80 µL of test compound or vehicle was added and J774.2 cells were incubated for 1 hour at 37°C (5% CO_2, 95% humidity). Lipopolysaccharide (LPS; 200 µg/mL LPS [Sigma-Aldrich Chemie GmbH] stock solution in phosphate-buffered saline [PBS; Sigma-Aldrich Chemie GmbH] diluted to 10 µg/mL with DMEM + 10% FBS) was added to the cells (80 µL/well) for a final LPS concentration of 1 µg/mL; instead of LPS, the no-trigger controls received 80 µL/well of DMEM + 10% FBS. Once LPS had been added, final concentrations were 0.03, 0.1, 0.3, 1, 3, 10, 30, and 100 µM for lefamulin and azithromycin, and 0.0003, 0.001, 0.003, 0.01, 0.03, 0.1, 0.3, and 1 µM for dexamethasone. All compounds were tested in duplicate, while trigger and no-trigger controls were tested in 4 replicas on every plate. After the compounds and vehicle were added, the plates were incubated for 1 hour at 37°C (5% CO_2, 95% humidity).

Cytokine/chemokine analysis

After incubation continued overnight, plates were centrifuged at 300×g for 10 minutes, and 750 µL supernatant was collected from each well for analysis of cytotoxicity and measurement of tumor necrosis factor (TNF)-α, interleukin (IL)-1β, IL-6, granulocyte-macrophage colony-stimulating factor (GM-CSF), chemokine (C-X-C motif) ligand (CXCL)-1, CXCL-2, chemokine (C-C motif) ligand (CCL)-2, and matrix metalloprotease (MMP)-9 concentrations via enzyme-linked immunosorbent assay (ELISA) using kits, antibodies, and standards from R&D Systems (Minneapolis, MN, USA). IL-1β and MMP-9 concentrations were assessed using the IL-1β/IL-1F2 and MMP-9, respectively, DuoSet ELISA kits per the manufacturer's protocols. For TNF-α, capture antibody was Human/Mouse TNF-α Antibody (AF-410-NA), detection antibody was Mouse TNF-α Biotinylated Antibody (BAF410), and standard was Recombinant Mouse TNF-α (aa 80-235) protein (410-MT). For IL-6, capture antibody was Mouse IL-6 Antibody (MAB406), detection antibody was Mouse IL-6 Biotinylated Antibody (BAF406), and standard was Recombinant Mouse IL-6 protein (406-ML). For GM-CSF, capture antibody was Mouse GM-CSF Antibody (MAB415), detection antibody was Mouse GM-CSF Biotinylated Antibody (BAF415), and standard was Recombinant Mouse GM-CSF protein (415-ML). For CXCL-1, capture antibody was Mouse CXCL1/GROα/KC/CINC-1 Antibody (MAB453), detection antibody was Mouse CXCL1/GROα/KC/CINC-1 Biotinylated Antibody (BAF453), and standard was Recombinant Mouse CXCL1/GROα/KC/CINC-1 (aa 20-96) protein (453-KC). For CXCL-2, capture antibody was Mouse CXCL2/GROβ/MIP-2/CINC-3 Antibody (MAB452), detection antibody was Mouse CXCL2/GROβ/MIP-2/CINC-3 Biotinylated Antibody (BAF452), and standard was Recombinant Mouse CXCL2/MIP-2 protein (452-M2). For CCL-2, capture antibody was Mouse CCL2/JE/MCP-1 Antibody (AF-479-NA), detection antibody was Mouse CCL2/JE/MCP-1 Biotinylated Antibody (BAF479), and standard was Recombinant Mouse CCL2/JE/MCP-1 protein (479-JE).

Immulon 2HB plates were coated with capture antibodies diluted in PBS (100 µL/well) and incubated at 4°C overnight. The next day, plates were washed 3 times with 300 µL/well of PBS (Sigma-Aldrich Chemie GmbH) + 0.05% Tween (Sigma-Aldrich Chemie GmbH) (PBS-T), 200 µL/well of blocking buffer was added (1% bovine serum albumin [BSA; Sigma-Aldrich Chemie GmbH] + 5% sucrose [Kemika, Zagreb, Croatia] in PBS-T) and incubated for 1 hour at 37°C.

Seven 2-fold serial dilutions of standards were prepared in 1% BSA in PBS-T. Supernatant samples were diluted in 1% BSA in PBS-T as follows: no dilution for MMP-9 and IL-1β analysis, 2-fold dilution for CXCL-1, 5-fold dilution for TNF-α and GM-CSF, 20-fold dilution for CXCL-2, 100-fold dilution for CCL-2, and 200-fold dilution for IL-6. Plates were washed, 100 µL/well of supernatant or standard/vehicle was added, and plates were incubated for 1 hour at 37°C. The plates were washed, detection antibody diluted in 1% BSA in PBS-T was added (100 µL/well), and plates were incubated for 45 minutes at 37°C. Plates were washed, 50 ng/mL streptavidin-HRP (horseradish peroxidase) in 1% BSA in PBS-T was added (100 µL/well), and plates were incubated for 30 minutes at 37°C. Tetramethylbenzidine (Sigma-Aldrich Chemie GmbH) substrate was added (100 µL/well); plates were incubated at room temperature (RT) and protected from light until a blue color developed in the wells (approximately 20 minutes). The reaction was stopped by adding 100 µL/well of 1 M sulfuric acid (Kemika). Absorbance at 450 nm was measured via Spectra Max i3 (Molecular Devices, San Jose, CA, USA).

Cytokine and chemokine levels in human PBMC supernatant

Human PBMC isolation

Human peripheral blood mononuclear cells (PBMCs) were isolated from buffy coat obtained from the Croatian Institute of Transfusion Medicine (CITM) from a healthy adult volunteer. The CITM ethics committee approved the blood collection process, and the volunteer provided written informed consent prior to blood collection. The buffy coat was diluted 1:1 with sterile PBS, and 25 mL of diluted buffy coat was layered over 20 mL of Lymphoprep (Axis-Shield Diagnostics, Ltd., Dundee, UK) and centrifuged for 35 minutes at 400×g (RT; acceleration and break turned off). Following centrifugation (300×g for 10 minutes at RT), the mononuclear ring was collected and transferred into a new 50-mL tube and washed 3 times with PBS; the remaining erythrocytes were lysed with an isotonic solution of ammonium chloride (150 mM; Kemika). The cell pellet was resuspended in Roswell Park Memorial Institute (RPMI) 1640 medium (Thermo Fisher Scientific) supplemented with 10% FBS (Sigma-Aldrich Chemie GmbH), and PBMCs were counted using a Sysmex XS-500i hematologic analyzer (Sysmex, Kobe, Japan). Cell concentrations were adjusted to 1.25×10^6 PBMCs/mL.

Treatment and stimulation of human PBMCs

Immediately after isolation from the buffy coat, 160 µL of PBMCs in RPMI 1640 + 10% FBS were seeded (200,000/well) in a 96-well plate. Border wells were filled with 200 µL of PBS. Freshly prepared 10× solutions of lefamulin, azithromycin, and dexamethasone were added (20 µL/well) to the plated PBMCs so that, after addition of LPS, final concentrations would be 0.3, 0.1, 1, 3, 10, 30, and 100 µM for lefamulin and azithromycin, and 0.0003, 0.001, 0.003, 0.01, 0.03, 0.1, 0.3, and 1 µM for dexamethasone. To the vehicle wells, 20 µL of 3% DMSO in medium was added so that the final DMSO concentration after LPS addition would be 0.3%. All compounds were tested in duplicate, while trigger and no-trigger controls were tested in 6 replicas on every plate. After addition of compounds and vehicle, plates were incubated for 1 hour at 37°C (5% CO_2, 95% humidity). LPS (200 µg/mL stock solution in PBS) was diluted to 1 µg/mL with RPMI 1640 + 10% FBS. After the cells had been incubated for 1 hour with the test compounds, 1 µg/mL LPS (20 µL/well) was added. The final concentration of LPS with the cells was 100 ng/mL. To the no-trigger controls, 20 µL/well of medium was added instead of LPS. Plates were incubated overnight at 37°C (5% CO_2, 95% humidity).

Cytokine/chemokine analysis

After overnight incubation, plates were centrifuged at 300×g for 10 minutes, and 180 µL of cell supernatant was collected from each well to be used for cytokine and cytotoxicity analyses.

To determine concentrations of IL-1β, GM-CSF, CCL-2, CXCL-1, and CXCL-2 in PBMC supernatants, a Luminex Human Magnetic Assay (R&D Systems) was used per manufacturer’s protocol. The plate was read using a Luminex 200 analyzer (Luminex Corporation, Austin, TX, USA) set to 50 events/bead, sample size 50 µL, and double discriminator gates at approximately 8000 and 16,500.

TNF-α, IL-6, and CXCL-8 concentrations in PBMC supernatants were above the quantification limit of the Luminex assay. Therefore, these cytokines, as well as MMP-9, were measured using ELISA kits, antibodies, and standards from R&D Systems. MMP-9 concentrations were assessed using Human MMP-9 DuoSet ELISA kit (DY911) per the manufacturer’s protocols. For TNF-α, capture antibody was Human TNF-α Antibody (MAB210), detection antibody was Human TNF-α Biotinylated Antibody (BAF210), and

standard was Recombinant Human TNF-α protein (210-TA). For IL-6, capture antibody was Human/Primate IL-6 Antibody (MAB206), detection antibody was Human/Primate IL-6 Biotinylated Antibody (BAF206), and standard was Recombinant Human IL-6 protein (206-IL). For CXCL-8, capture antibody was Human IL-8/CXCL8 Antibody (MAB208), detection antibody was Human IL-8/CXCL8 Biotinylated Antibody (BAF208), and standard was Recombinant Human IL-8/CXCL8 protein (208-IL). ELISAs followed the same procedures described above for measurement of cytokine and chemokine levels in J774.2 mouse macrophage supernatants, except that PBMC supernatant samples were diluted 50-fold for TNF-α analysis, 150-fold for IL-6, 800-fold for CXCL-8, and 5-fold for MMP-9.

Cytotoxicity evaluation

To evaluate cytotoxicity, 5 μL of supernatant from treated and stimulated J774.2 mouse macrophages or human PBMCs were mixed with 25 μL of Adenylate Kinase Detection reagent (ToxiLight Non-destructive Cytotoxicity BioAssay Kit; Lonza, Basel, Switzerland) in a white Lumitrac 384-well plate. Plate was incubated for 5 minutes in the dark, after which luminescence was measured by use of EnVision 2104 Multilabel Plate Reader (PerkinElmer, Waltham, MA, USA).

Statistical analysis

Concentrations of all measured cytokines were determined by interpolation from their standard curves. For each compound, percentage of inhibition compound was calculated from the obtained concentrations using the formula:

$$\left(1-\frac{(test\ compound-average\ no\ trigger)}{(average\ trigger-average\ no\ trigger)}\right)\times 100$$

The effect of test compounds on cell viability of J774.2 macrophages and human PBMCs was determined by comparing the adenylate kinase signal obtained in the presence of different concentrations of compounds with those obtained in DMSO (vehicle) control. Signals were divided, and the results were expressed as fold-change versus vehicle control.

Supplemental Figure 1. (A) Total and (B) neutrophil cell counts in BALF after LPS induction.

BALF, bronchoalveolar lavage fluid; Dexa, dexamethasone; LPS, lipopolysaccharide; PO, oral; SC, subcutaneous. Box and whisker plots show 25% percentile, median, and 75% percentile in box, with minimum and maximum values shown with whiskers. Means are shown with "+" and raw data points with black circles. In the Dexa group, 1 animal did not recover from anesthesia after LPS challenge. Because no samples from this animal were available for collection, the Dexa group included 7 animals; all other groups included 8 animals. **P*<0.05 vs LPS/vehicle via Mann-Whitney test.

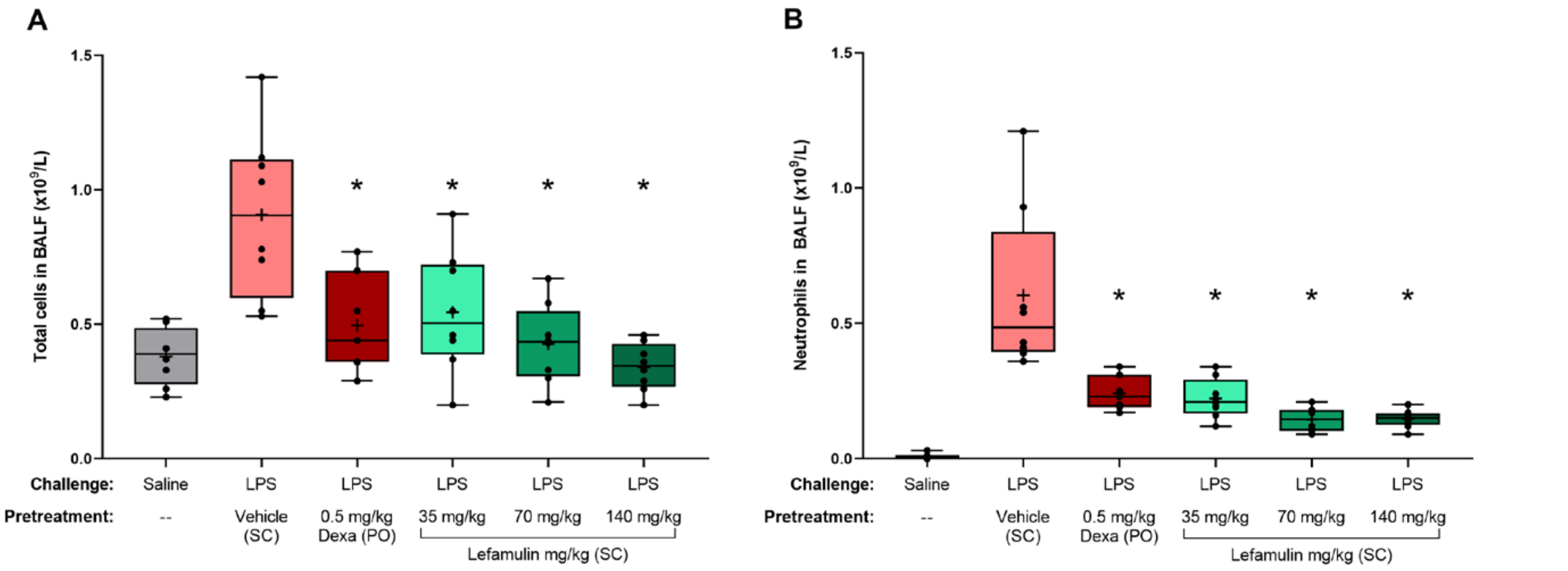

Supplemental Figure 2. LPS-induced cytokines and chemokines in J774.2 mouse macrophage cell supernatant.

CCL, chemokine (C-C motif) ligand; CXCL, chemokine (C-X-C motif) ligand; GM-CSF, granulocyte-macrophage colony-stimulating factor; IL, interleukin; LPS, lipopolysaccharide; MMP, matrix metalloprotease; TNF, tumor necrosis factor.

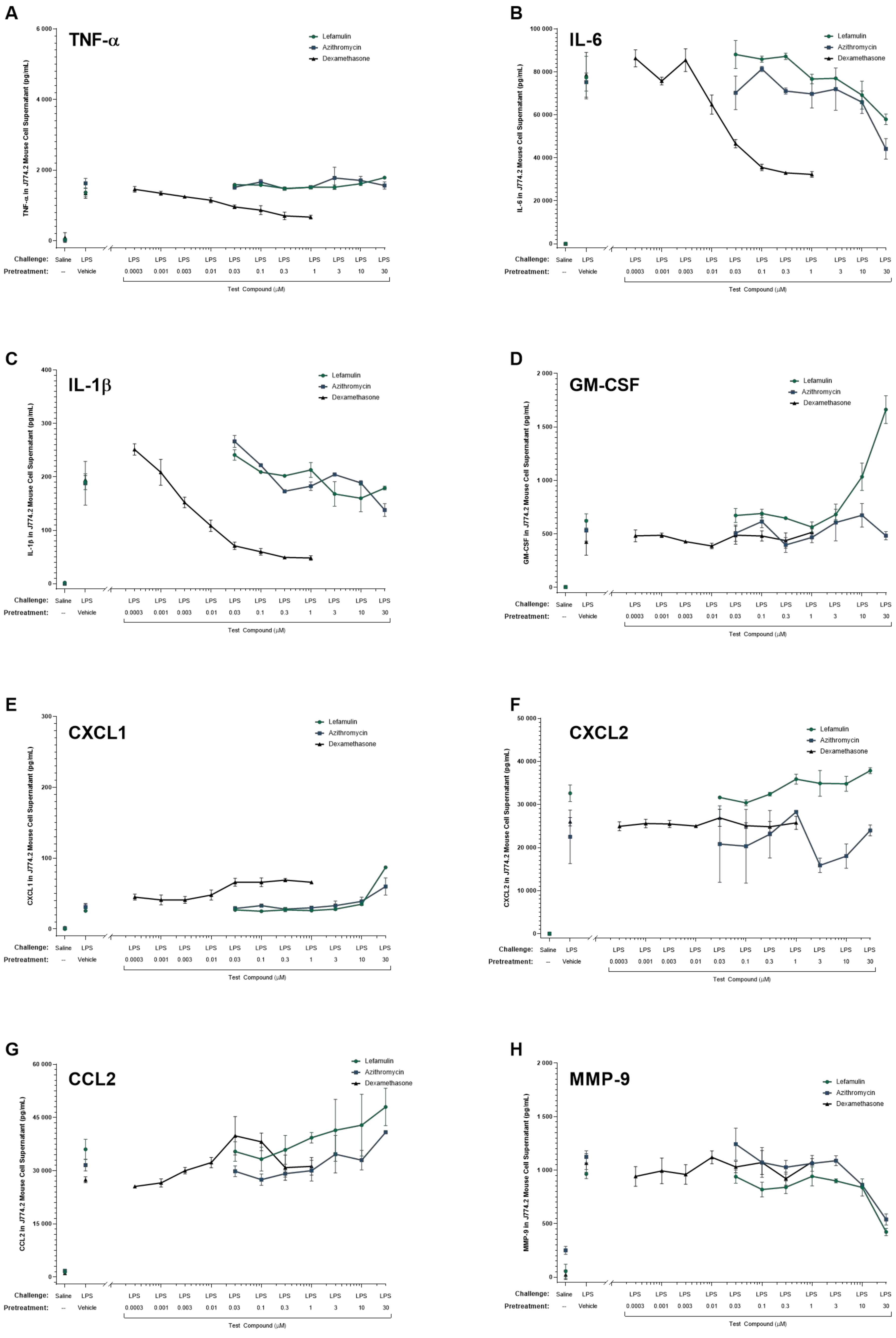

Supplemental Figure 3. LPS-induced cytokines and chemokines in human PBMC supernatant.

CCL, chemokine (C-C motif) ligand; CXCL, chemokine (C-X-C motif) ligand; GM-CSF, granulocyte-macrophage colony-stimulating factor; IL, interleukin; LPS, lipopolysaccharide; MMP, matrix metalloprotease; PBMC, peripheral blood mononuclear cell; TNF, tumor necrosis factor.

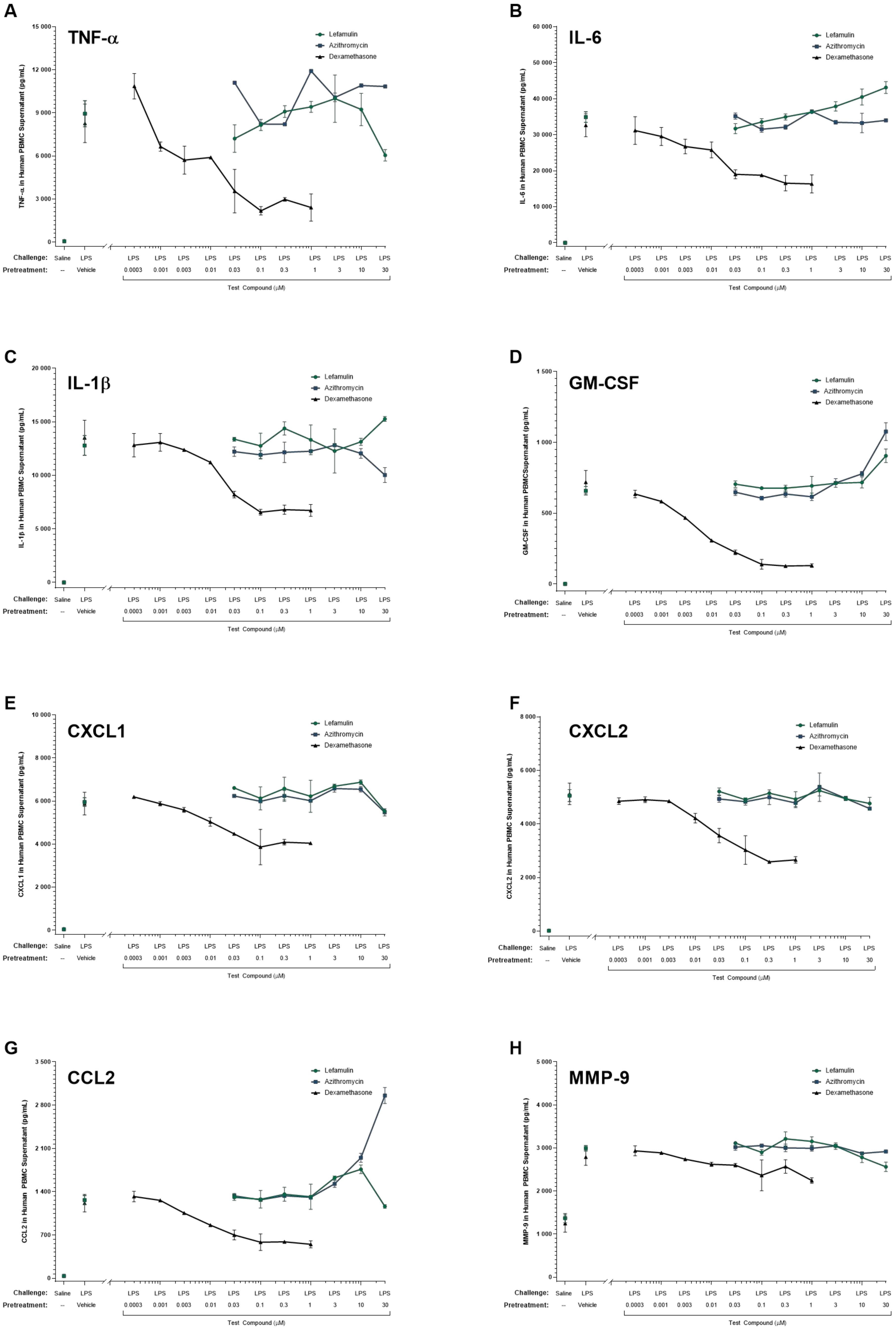

Supplemental Table 1. Cytotoxic effects of lefamulin and azithromycin on human neutrophils, J774.2 mouse macrophages, and human PBMCs*

	Mean ± SEM							
	**0.03 µM**	**0.1 µM**	**0.3 µM**	**1 µM**	**3 µM**	**10 µM**	**30 µM**	**100 µM**
**Human neutrophils, % viability† at test concentration vs vehicle control**								
Lefamulin	105 ± 1.36	106 ± 1.34	105 ± 2.39	103 ± 4.22	103 ± 3.07	102 ± 2.98	101 ± 2.58	86 ± 2.99
Azithromycin	105 ± 0.70	106 ± 1.01	106 ± 1.25	106 ± 0.33	105 ± 1.81	107 ± 2.01	106 ± 1.74	106 ± 2.20
**J774.2 mouse macrophages, fold-change of adenylate kinase release at test concentration vs vehicle control**								
Lefamulin	1.33 ± 0.11	1.27 ± 0.23	1.10 ± 0.16	0.89 ± 0.08	1.12 ± 0.18	1.26 ± 0.27	2.00 ± 0.13	6.18 ± 0.05
Azithromycin	1.13 ± 0.03	0.90 ± 0.07	0.81 ± 0.08	0.87 ± 0.11	1.11 ± 0.16	1.22 ± 0.01	1.44 ± 0.10	2.65 ± 0.24
**Human PBMCs, fold-change of adenylate kinase release at test concentration vs vehicle control**								
Lefamulin	1.07 ± 0.03	1.04 ± 0.01	1.03 ± 0.02	1.00 ± 0.01	0.95 ± 0.01	0.93 ± 0.01	0.92 ± 0.03	1.20 ± 0.03
Azithromycin	1.02 ± 0.02	1.02 ± 0.01	0.99 ± 0.01	0.97 ± 0.01	0.93 ± 0.01	0.90 ± 0.02	0.83 ± 0.01	0.93 ± 0.03

PBMC=peripheral blood mononuclear cell; RLU=relative light unit.

*At each dose, J774.2 mouse macrophages and human PBMCs were tested in duplicate (n=2), and human neutrophils were tested in triplicate (n=3).

†Assessed via percentage of RLU.
